# Identification and validation of core genes as promising diagnostic signature in hepatocellular carcinoma based on integrated bioinformatics approach

**DOI:** 10.1038/s41598-022-22059-6

**Published:** 2022-11-09

**Authors:** Pradeep Kumar, Amit Kumar Singh, Kavindra Nath Tiwari, Sunil Kumar Mishra, Vishnu D. Rajput, Tatiana Minkina, Simona Cavalu, Ovidiu Pop

**Affiliations:** 1 Department of Botany, MMV, Banaras Hindu University, Varanasi, Uttar Pradesh 221005 India; 2 Department of Pharmaceutical Engineering and Technology, Indian Institute of Technology, Banaras Hindu University, Varanasi, Uttar Pradesh 221005 India; 3 Academy of Biology and Biotechnology, Southern Federal University, Rostov-on-Don, Russia; 4 Faculty of Medicine and Pharmacy, University of Oradea, P-Ta 1 Decembrie 10, 410087 Oradea, Romania

**Keywords:** Cancer, Computational biology and bioinformatics

## Abstract

The primary objective of this investigation was to determine the hub genes of hepatocellular carcinoma (HCC) through an in silico approach. In the current context of the increased incidence of liver cancers, this approach could be a useful prognostic biomarker and HCC prevention target. This study aimed to examine hub genes for immune cell infiltration and their good prognostic characteristics for HCC research. Human genes selected from databases (Gene Cards and DisGeNET) were used to identify the HCC markers. Further, classification of the hub genes from communicating genes was performed using data derived from the targets' protein–protein interaction (PPI) platform. The expression as well as survival studies of all these selected genes were validated by utilizing databases such as GEPIA2, HPA, and immune cell infiltration. Based on the studies, five hub genes (*TP53*, *ESR1*, *AKT1*, *CASP3*, and *JUN*) were identified, which have been linked to HCC. They may be an important prognostic biomarker and preventative target of HCC. In silico analysis revealed that out of five hub genes, the *TP53* and *ESR1* hub genes potentially act as key targets for HCC prevention and treatment.

## Introduction

Non-communicable diseases include brain haemorrhage, severe pulmonary illnesses, cardiovascular disease, insulin resistance, and carcinoma. These are the leading causes of death globally^1^. The cause of death might be due to both internal and external reasons. In the recent report^2^, among all the cancers, hepatic carcinoma is among the most probable causes of cancer. Regarding the total deaths reported globally, HCC (hepatocellular carcinoma) is the third major cause of death. A total of 906,000 cancer cases were reported worldwide. In these cases, mortality occurred in 830,000 cases^2^. Among the liver cancer patients, 75–85% of patients are affected by HCC, while 10–15% of patients are suffering from intra-hepatic cholangiocarcinoma. Viral hepatitis B or C infection, aflatoxin-based food contaminants, excessive alcohol consumption, weight gain, type 2 diabetes, and tobacco consumption are the key leading causes of HCC. The leading causes of HCC in the world are region-dependent^2^. Liver cirrhosis is a major hepatic disease. It is also identified as a major health risk for HCC^3^. Clinically, in liver cirrhosis, the progressive deterioration of the hepatic functions takes place, which leads to HCC development^4^.

Despite the availability of modern clinical facilities such as surgical removal, organ transplants, catheter-based arterial chemoembolization, and radiofrequency ablation, patients with HCC have a low chance of survival^5^. Modern therapeutic techniques for liver cancer have improved incidence and morbidity. However, HCC survival rates are quite poor in the early stages^6^. In general, clinically significant signs and symptoms of HCC are not recognized in the early stages of cancer. Thus, in these stages, treatment was missed for cancer complications^7^. Patients with HCC can benefit from timely and successful therapy in terms of both wellbeing and life longevity^8,9^. Consequently, the emergence of new prognostic potential signatures and therapeutic targets are crucial concerns in the treatment of HCC. Previous studies have shown that valuable prognostic biomarkers, like functional genes, were recognized through bioinformatics analysis, which is based on the high-throughput data of HCC^10–12^. Computational approaches could be used to analyze the gene expression datasets, which revealed the important mechanisms of action of the genes involved in HCC treatment^13^.

In the present work, we have identified the prevalent HCC genes from the Gene Card and DisGeNET datasets. Further, protein–protein interaction (PPI) with these prevalent genes was performed. In this study, hub genes were selected based on ontological characteristics such as degree of closeness, betweenness, etc. Then, Gene Ontology (GO) evaluation and the Kyoto Encyclopaedia of Genes and Genomes (KEGG) pathway linked to HCC were investigated. The TIMER2.0 database is utilized for the evaluation of potential links between each hub gene and the infiltrated immune cells. Databases, such as GEPIA2^14^, HPA^15^, and TIMER2.0^16^, were used to visualize the prognostic scenario of candidate hub genes during the present investigation. This technique was employed to ensure a stable platform for exploring new biomarkers for disease diagnosis and to find potential treatment targets for HCC. Hub genes were screened in this work by merging the results from the PPI and GO datasets. Finally, expression and survival analysis and immune infiltration analysis were used to validate the hub genes of HCC. Thus, in the present study, hub genes of the HCC were identified through an in-silico approach.

## Results

### Identification of target gene

**Figure 1:**
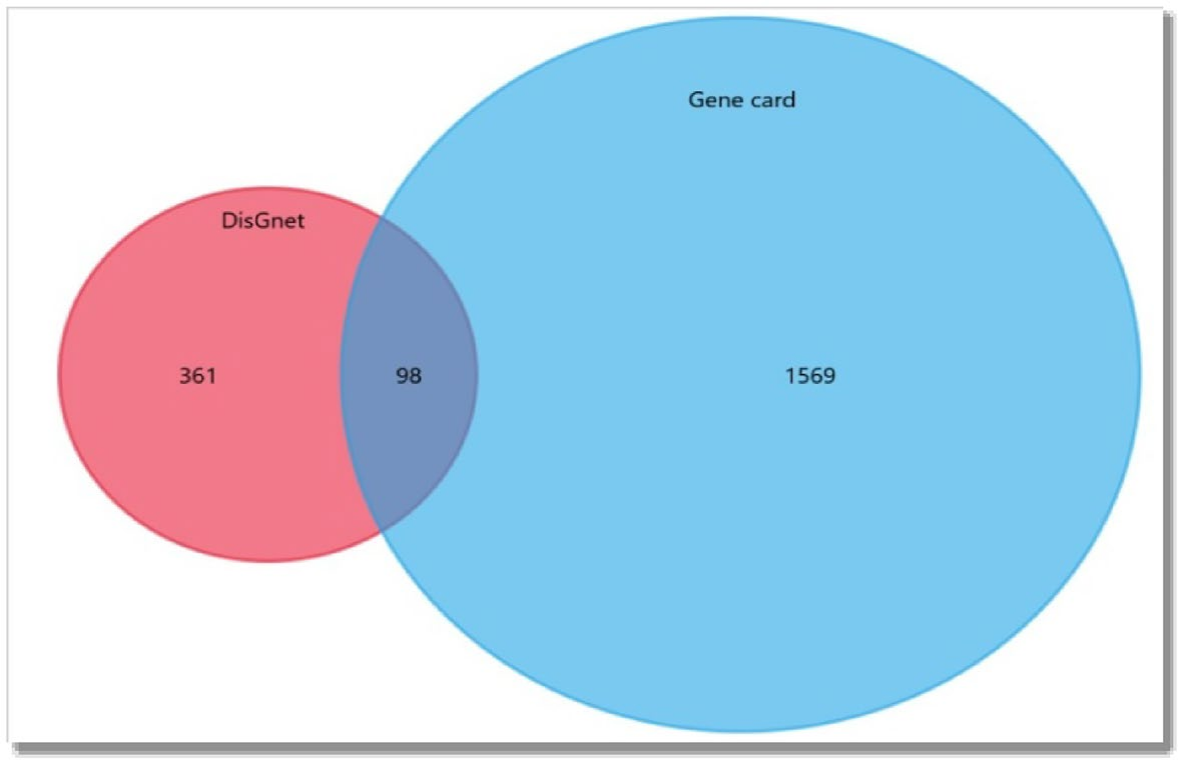
Venn diagram showing the target genes for HCC based on different dataset.

From the datasets, a total of 1569 HCC target genes from Gene Card and 361 genes from DisGenet were taken. In the present study, 98 prevalent genes were retrieved and further recognized from datasets for network design (Fig. 1).

### PPI network building and hub gene identification

**Figure 2:**
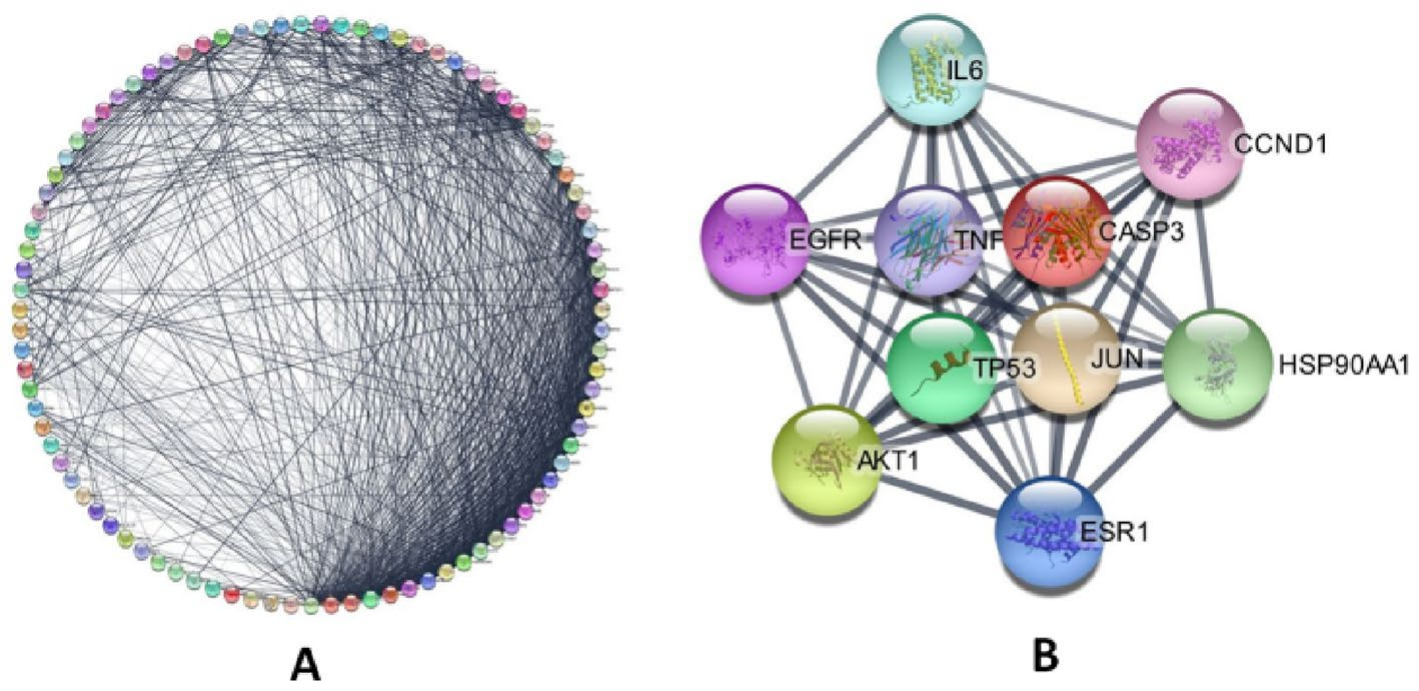
PPI network showing the distribution of nodes and edges (**A, B)** which attributes the presence of hub gene Network (https://string-db.org/).

Using the STRING database, a PPI network was created based on 98 common genes. Results revealed that 91 nodes as well as 1068 edges with a 0.695 clustering coefficient along with an enrichment p-value < 1.0E−16 were recorded (Fig. 2A). Based on the degrees of connectivity, the top ten genes were screened through CytoNCA. These genes were represented as hub genes. Based on the degree, five interlinked genes (*TP53*, *ESR1*, *AKT1*, *CASP3*, and *JUN*) were selected for further investigation (Fig. 2B).

### Functional annotation of hub genes

**Figure 3:**
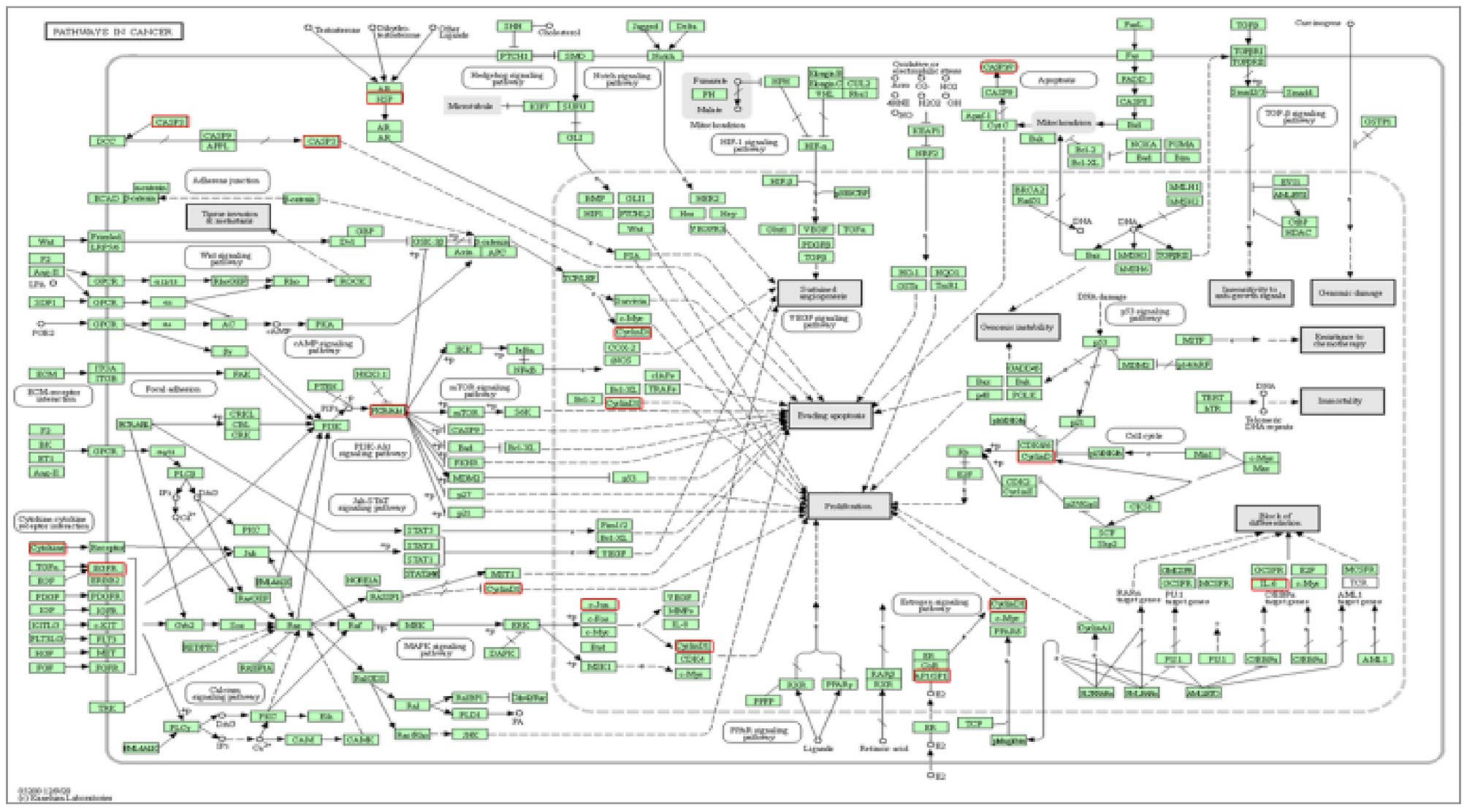
The possible role of hub gene in the regulation of HCC pathways (https://www.genome.jp/kegg).

On the basis of the GO study and gene enrichment analysis with the KEGG pathway, it is clear that these hub genes regulate the different bioactivities as shown in Table S1. These bioactivities are concerned with different molecular functions (MF), biological processes (BP), as well as cellular components (CC) (Table S1). KEGG analysis revealed the involvement of the hub genes in different signaling pathways such as estrogen, FoxO, IL-17, cancer pathways, mTOR, JAK-STAT, VEGF, PI3K-Akt, Ras, and Toll-like receptor (Table S2). Information based on the bioactivities and signaling pathways, the possible role of these hub genes in cancer regulation processes is shown in Fig. 3.

### Assessment of hub genes survival

**Figure 4:**
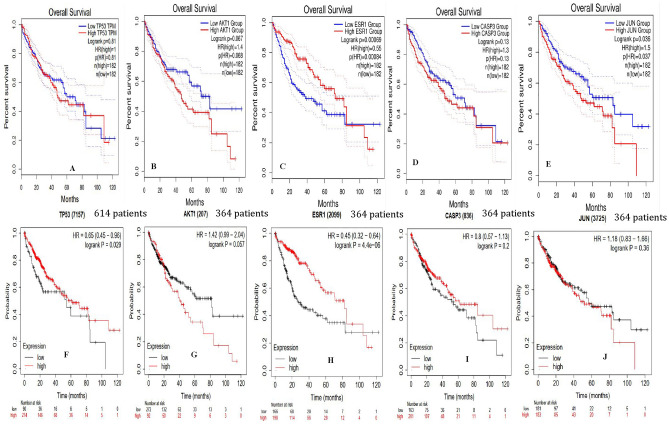
The overall survival of patients (**A–E**) and (**F–J**) with HCC by Kaplan–Meier plots (https://kmplot.com/analysis).

An overall survival study of hub genes was carried out to describe the identification of prognostic variables in selected five hub genes for HCC. The Kaplan–Meier plotter and GEPIA2 analysis revealed significantly (p > 0.05) higher expressions of hub genes *TP53* and *JUN* in patients with HCC. Similarly, the *ESR1* hub gene displayed significantly (p < 0.05) lower expression, as confirmed during GEPIA2 and Kaplan–Meier Plotter analysis, in patients suffering with HCC (Fig. 4). Thus, we observed that these hub genes were poorly linked with prognosis in HCC patients. However, hub genes *AKT1* and *CASP3* were not utilized for further analysis due to variables showing violation (p > 0.05) in the GEPIA2 database as well as results obtained through Kaplan–Meier Plotter analysis.

### Verification of hub gene mRNA and protein expression

**Figure 5:**
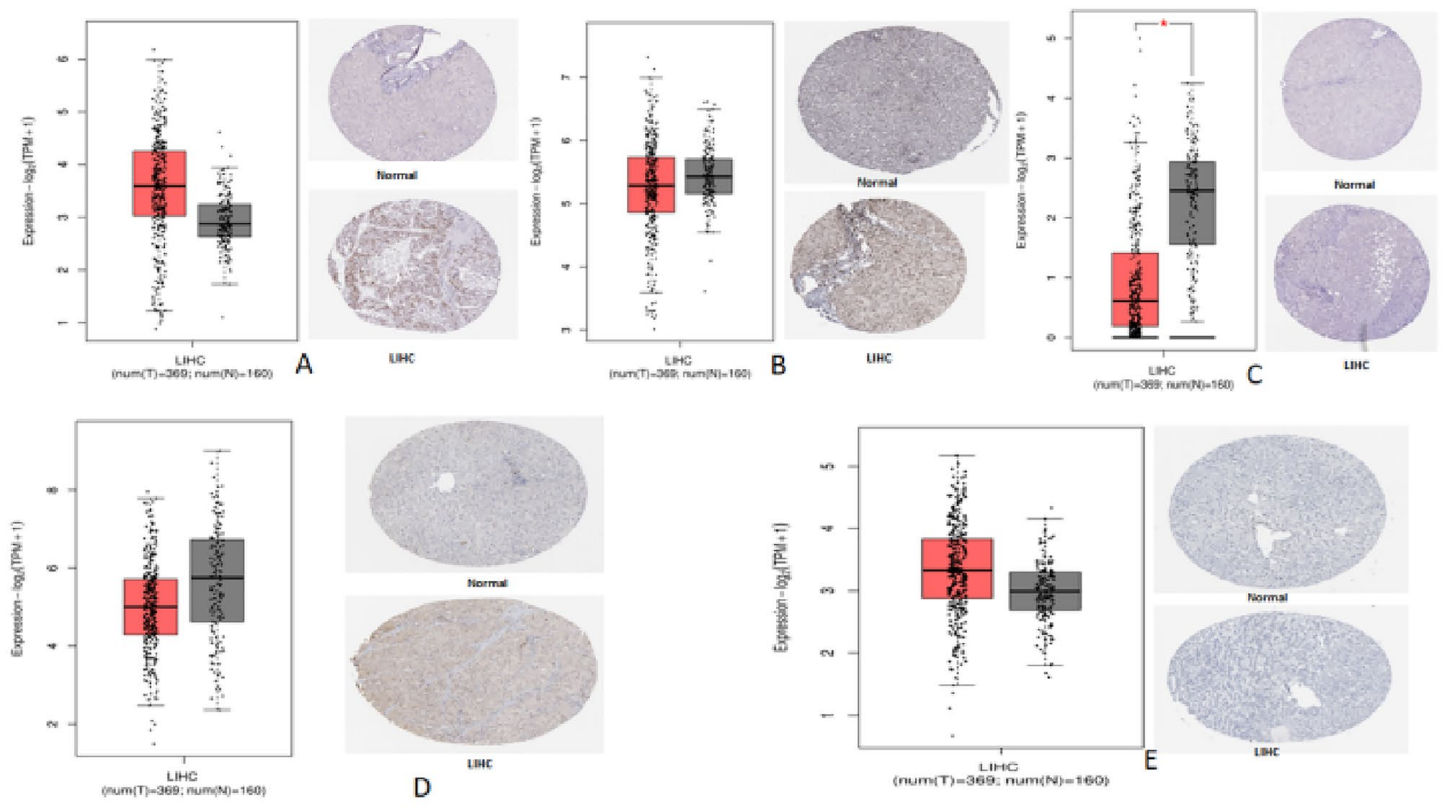
The mRNA expression and immunohistochemistry of the (**A)** *TP53*, (**B)** *AKT1*, (**C)** *ESR1*, (**D)** *JUN* and (**E)** *CASP3* in normal liver tissues and HCC tissues from the GEPIA2 (http://gepia2.cancer-pku.cn/#index) and HPA database (https://www.proteinatlas.org/). The gray bars in box plots represent normal samples; the red bars in box plots represent tumor samples(*p < 0.01).

The GEPIA2 database was used to validate the mRNA expression levels of selected five hub genes in normal and HCC samples (Fig. 5A–E). *ESR1* expression was significantly higher in HCC thanin normal hepatic tissues (Fig. 5C). Similar results were reported by several researchers^17^.

HPA immune histochemistry data revealed that *TP53* and *CASP3* protein expressions were positively up-regulated in HCC tissues compared to normal (Fig. 5A,D), whereas hubgenes *AKT1*, *ESR1*, and *JUN* protein expressions were negatively down-regulated in HCC tissues compared to normal (Fig. 5B,C,E). Thus, the mRNA expressions of genes as well as protein expression of the selected five hub genes were regulated in patients suffering with HCCin comparison to normal liver tissue.

**Figure 6:**
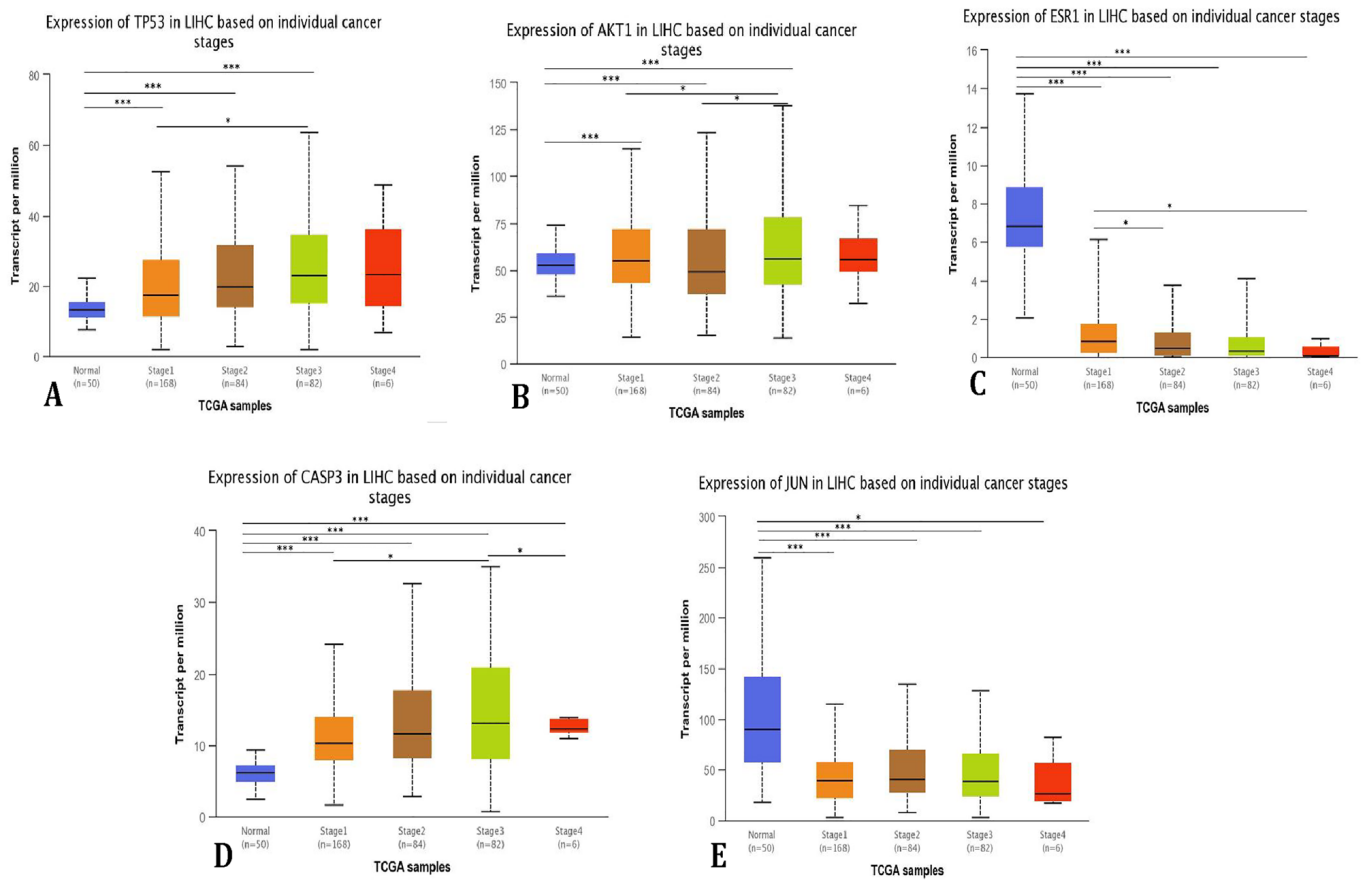
The mRNA expression of the *TP53* (**A**), *AKT1* (**B**), *ESR1*(**C**), *JUN*(**D**) and *CASP3* (**E**) in individual cancer(*p < 0.05, ***p < 0.001) (http://ualcan.path.uab.edu).

The UALCAN database was used for the expression correlation analysis of selected five hub genes linked with HCC. The results of the correlation revealed the significant expression of the selected genes at different stages of HCC (Fig. 6A–E). Based on the findings, hub genes *TP53*, *ESR1*, *AKT1, CASP3*, and *JUN* were compactly regulated with the risk of HCC development and its progression chronically.

### Hub gene expression in various immune cells

**Figure 7:**
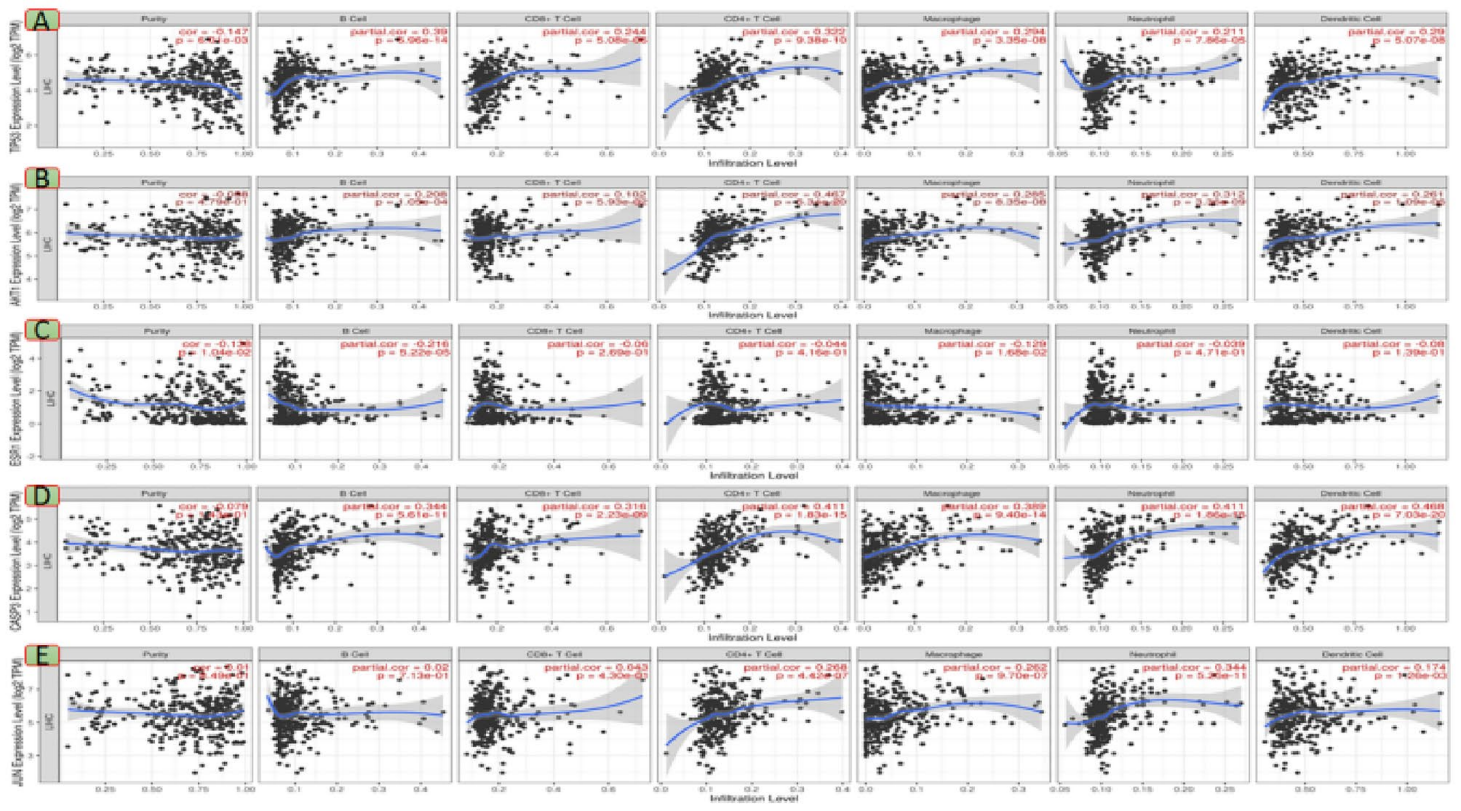
Analysis of hub gene expression in various immune cells in HCC.*TP53* (**A**), *AKT1*(**B**), *ESR1*(**C**), *CASP3* (**D**) and *JUN* (**E**) (http://timer.cistrome.org).

**Figure 8:**
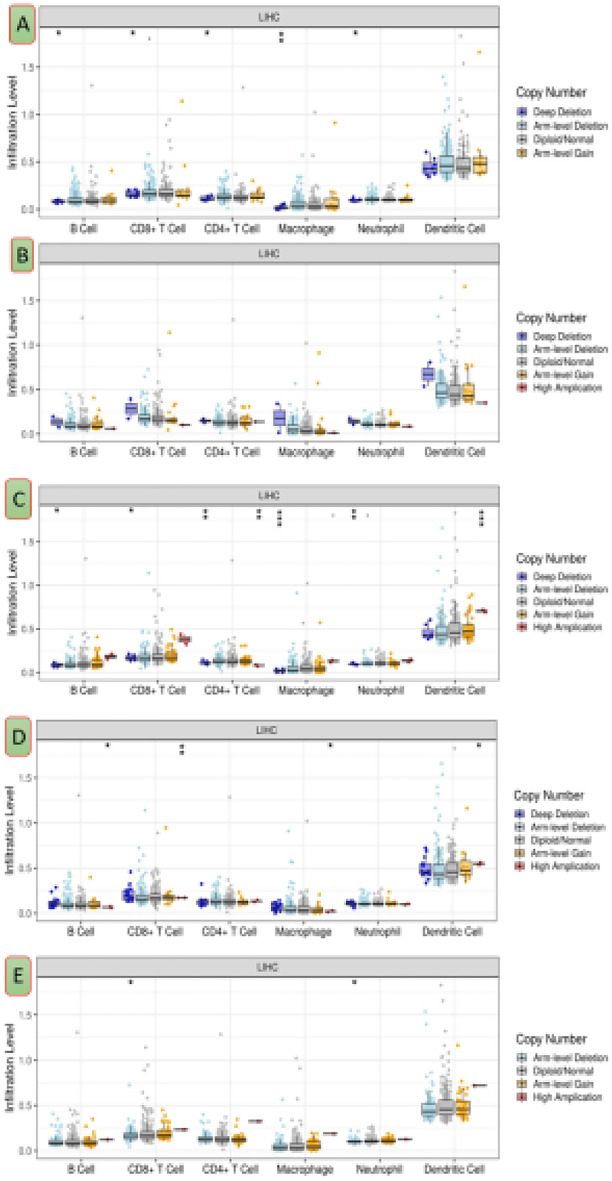
Hub gene expression analysis in different immune cells (http://timer.cistrome.org).

The TIMER database results revealed that the *TP53* and *ESR1* genes have a significant correlation with the tumor purity parameter. The expression of hub genes (*AKT1*, *ESR1*, and *JUN*) was down regulated and correlated against tumor-infiltrating levels negatively into LIHC; in contrast, up regulated hub genes (*TP53* and *CASP3)* expression was correlated against all TIICs subsets positively into HCC (Fig. 7 A,E). In LIHC, *AKT1* gene expression (Fig. 7B)was significantly correlated with five TIICs except CD8+ cells*.* The infiltration of B cells and macrophages in *ESR1* gene expression (Fig. 7C) was significantly observed. *JUN* gene expression was significant against dendritic cells (DCs), CD4+ T cells, neutrophils, and macrophage infiltration levels (Fig. 7E). In comparison to DCs, neutrophils, and CD8+ T cells (Fig. 8), the hub genes *TP53* and *CASP3* (Fig. 8A,E) were significantly more expressed in macrophages, CD4+ T cells, and B cells. Results suggested that macrophages have a preferential corner in the immune infiltration study in HCC with reference to selected five hub genes.

## Discussion

The International Agency for Research on Cancer (IARC) documented that liver cancer diseases are the 6th most commonly diagnosed cancer, while they were the 3rd major cause of global mortality during 2019–2020^18^. Non-alcoholic steatohepatitis is also one of the most prevalent and predominant liver cancers^19,20^. The identification of hub genes of HCC significantly expanded the research in the areas of bioactivities, survival analysis, expression, and poor prognosis values, as well as immune infiltration using bioinformatics approaches.

The expression of 91 overlapping genes varied in HCC tissues. Furthermore, the hub genes were chosen for their high degree of connectivity. Hub gene expression verification, overall survival, as well as immune infiltration were analyzed. According to the DAVID database, compromised genes were significantly associated with various signaling pathways that were linked with HCC. The GEPIA database was used to validate that *TP53* and *CASP3* mRNA expression were significantly higher. *TP53* is an essential mediator of apoptosis in numerous cell types*.* It is evidenced by an increase in *TP53* expressions. *TP53* has the ability to regulate the expression of target genes that are involved in multiple activities, including cell cycle arrest, DNA repair, apoptosis, and preventing deformation processes. *TP53* is also important for mitochondrial trans-membrane stability, and it increases apoptosis without transcriptional regulation. The *BAX* gene promoter area can be bound with wild-type *TP53*, which regulates the expression of the *BAX* gene^21,22^. It was observed that *AKT1*, *ESR1*, and *JUN* mRNA thresholds were significantly lower in expression in HCC patients.

*BAX* is a *BCL2* family member that causes apoptosis^23^. *BAX* is a *TP53* target. *The BAX* constructs complexes of *BCL2* and inhibits its function while promoting the function of cytochrome c in the mitochondria initially and later into the cytoplasmic matrix. Thus, it aids *caspase* activation and apoptosis implementation. *CASP3* is a key player in apoptotic and necrotic cell death through intrinsic and extrinsic pathways. ROS production and apoptotic cell detachment are also inhibited by *CASP7* and *CASP9*.These caspases support the apoptotic pathways of *CASP3*. The TP53-BAX-caspase3 signaling pathway induced apoptosis of HCC cells by up-regulating *TP53 *and activating *BAX* and *CASP3 *protein expression^24^.

The outcome of the hub gene results revealed that abnormal miRNAs play a pivotal function in the development and progression of HCC. Application of miRNAs has recently been introduced as a diagnostic, curative, and prognostic biomarker^25^. *AKT1* is believed to play an important role in HCC oncogenesis, and it has been identified as a key target of most miRNAs in the disease. miRNAs suppressed the expression of key hub genes related to cancer. It suggests that miRNAs may be useful in the detection and therapeutic strategies for the control of HCC. *AKT1* is a critical receptor for the activation of the highly cancer-causing Wnt/β-catenin signaling pathway, which is linked with cancer development. This signalling pathway is also connected to HCC uncontrolled cell proliferation^26^.

Activating protein 1 is a protein family, incorporating *JUN*, which is a dimer and also functions as a transcription factor. Multiple downstream genes are regulated by such proteins in genetic transcription processes after attachments with the DNA strand. It is responsible for the regulation of cell proliferation, differentiation, invasion, and metastasis etc. Thus, it was clear that activating protein1 plays an important role in cancer development^27^. *JUN* is found in both homodimerized and heterodimerized forms in the AP-1 family^27^. The heterodimers of *JUN* have more stable and relatively stronger DNA-binding behaviour than homodimers of *JUN*^28^. According to accumulating evidence, v-JUN has been one of the 17 modifying oncogenes of the bird malignancy virus and is cellularly homologous to *JUN*^29^*. JUN* appears to be important in the tumour development and growth of HCC^28^. Its higher expression has been reported in human hepatocellular carcinoma cells^27^. Furthermore, data from different experimental models with reduced expression of *JUN* revealed that the involvement of *JUN* in HCC signaling pathways, which influenced cancer cell proliferation and migration^30^.

The observation of HCC tissues revealed a significantly lower expression of *ESR1* mRNA. It caused the down-regulated expression of *ESR1*. Inhibition of *ESR1* expression could significantly neutralize the inhibition activity of miRNA, which leads to suppression of HCC cell development and progression. The function of miRNAs is important in promoting cell proliferation, migration, and invasion. Thus, targeting *ESR1-based* treatment of patients suffering from HCC can successfully bring wellness to these patients^31^.

*ABCC5*^32^, hub gene, and immune subtype prognostic biomarkers^33^ have all been linked to HCC. *ABCC5* is involved inseveral mechanisms liketrans-membrane transport as well as immune cell filtration of HCC.

In-silico analysis revealed the association of five hub genes (*TP53, ESR1, AKT1, CASP3*, and *JUN*) with HCC. These hub genes may be used as liver cancer prognostic biomarkers. The role of these hub genes as biomarkers is based on the analysis of PPI, KEGG pathways, miRNAs, protein expression, and immune cell filtration. This information is based on bioinformatics approaches, which may require additional in vitro and in vivo experimental validation before concluding their exact molecular mechanisms.

## Conclusion

HCC is a global health problem that affects millions of people each year. The primary goal of this work was to highlight the relevant hub genes identification, which are involved in HCC. It will be helpful in the risk assessment of HCC and also in the prevention approach. Results revealed that the mRNA and protein thresholds of the five hub genes are associated with several important signaling pathways, which are significantly diagnosed in HCC patients. Based on the data related to the identification, level of expression, and survival patterns of the five hub genes in HCC, our study provides significant information with relevance for the management and treatment of HCC patients.

## Methods

### Data mining

The relevant HCC targets were obtained from the human gene directory GeneCards (https://www.genecards.org/)^34^ and DisGeNET (Version 7.0) (https://www.disgenet.org/)^35^. The parameter for selecting disease target genes in this analysis of the data was "score" ≥ mean score.

### Data processing and target screening

The Venn chart was created with the FunRich software^36^ to screen for intersecting genes between GeneCards and DisGeNET database genes of HCC. The intersection of anticipated targets of both databases was used to obtain the targets of the HCC.

### Generation of protein protein interaction (PPI) networks and recognition of hub genes

The PPI network was first plotted using the online Search Tool for the Acquisition of Interacting Genes (STRING)^37^ (https://string-db.org/). It was further displayed and interpreted through Cytoscape software^38^. The CytoNCA was also used to find the hub genes with the highest degree of association, which have been filtered with regard to degree, closeness, or betweenness.

### Investigations of candidate genes enrichment and pathway

This analysis was performed with the help of GO and KEGG pathways. GO is a functional procedure that helps in the classification of hub genes on the basis of biological processes, molecule functioning, and cellular comportment, while the KEGG^39^ (https://www.genome.jp/kegg) and DAVID databases (https://david.ncifcrf.gov/) help in the enrichment analysis for mechanism of action and to analyze the possible function of the preferred hub genes^40,41^.

### Verification and survivability assessment of hub genes

The GEPIA2 database was selected to evaluate the impact of hub gene biomarkers on HCC patient survival rates. It depicts the analysis of normal and tumour sample data, which were collected from TCGA and GTEx^42^. The genes found significantly (p < 0.05) in the formats were chosen for further investigation. The biomarkers were translated into expression profiles for selected hub genes in normal as well as HCC samples and further visualized and evaluated using GEPIA2.

In addition, for protein expression in the human protein atlas, a web interface (http://www.proteinatlas.org) has been utilized to assess the role of genes in normal as well as HCC samples. Subsequently, another interface, UALCAN(http://ualcan.path.uab.edu/), was used for the examination of relationships between different stages of cancer for mRNA expression of the genes of interest using the TCGA set of data^43^.

### Immune infiltration

TIMER is a cancer-targeted web-accessible immune infiltration database, which is a robust and efficient database that analyzes the immune cell infiltration (TIMER 2/) by utilizing data from TCGA from 32 different cancers^40^. The tumor-infiltrating immune cells (TIICs) comprise CD4+ T cells, dendritic cells (DCs), macrophages, CD8+ T cells, B cells, and neutrophil cells. TIMER was used for the correlation of gene expression between immune cells and hub genes.

### Ethics declarations

All methods were carried out in accordance with relevant guidelines and regulations.

## Supplementary Information

Supplementary Tables.

## Data Availability

The datasets used and/or analyzed during this study are available from the corresponding author on reasonable request.
