# Supplementary material for: Identification and validation of core genes as promising diagnostic signature in hepatocellular carcinoma based on integrated bioinformatics approach
Source: Sci Rep. 2022 Nov 9;12:19072. doi: 10.1038/s41598-022-22059-6 (PMC9646875; doi:10.1038/s41598-022-22059-6)
Supplement: Supplementary file 1 — Supplementary Tables. [file 41598_2022_22059_MOESM1_ESM.docx]

# **
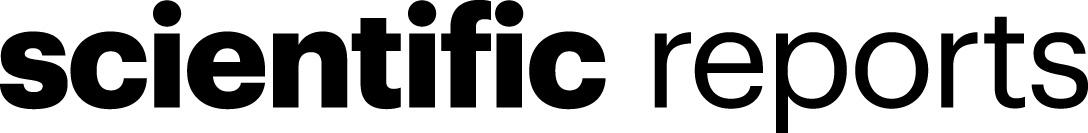
**

**Identification and validation of core genes as promising diagnostic signature in hepatocellular carcinoma based on integrated bioinformatics approach**

Pradeep Kumar^1^, Amit Kumar Singh^2^, Kavindra Nath Tiwari^1#^, Sunil Kumar Mishra^2^, Vishnu D Rajput^3^, Tatiana Minkina^3^, Simona Cavalu^4#^, Ovidiu Pop^4^

^1^ Department of Botany, MMV, Banaras Hindu University, Varanasi, Uttar Pradesh, 221005, India

^2^ Department of Pharmaceutical Engineering & Technology, Indian Institute of Technology, Banaras Hindu University, Varanasi, Uttar Pradesh 221005, India

^3^ Academy of Biology and Biotechnology, Southern Federal University, Rostov on Don, Russia

^4^ Faculty of Medicine and Pharmacy, University of Oradea, P-ta 1 Decembrie 10, 410087 Oradea, Romania

* Corresponding author: Prof. Kavindra Nath Tiwari ([kntiwaribhu@gmail.com](mailto:kntiwaribhu@gmail.com) ; [kntiwari@bhu.ac.in](mailto:kntiwari@bhu.ac.in); ORCID: 000-0002-8951-9057). Simona Cavalu (Simona.cavalu@gmail.com)

**Supplementary materials**

**Table captions**

**Table S1.** Gene ontology-based bioactivities regulated by hub genes.

**Table S2.** Role of hub genes in signaling pathways as analyzed by KEGG.

| **Parameters** | **Category ID** | **Bioactivities** | **p-value** | **Hit in Query list** |
| --- | --- | --- | --- | --- |
| Biological properties | BP(GO:1903798) | Regulation of production of miRNAs involved in gene silencing by miRNA | 9.607e-11 | IL6, TNF, EGFR, ESR1 |
|  | BP(GO:0045737) | Positive regulation of cyclin-dependent protein serine/threonine kinase activity | 6.098e-8 | CCND1, AKT1, EGFR |
|  | BP(GO:0050999) | Regulation of nitric-oxide synthase activity | 2.570e-9 | HSP90AA1, TNF, AKT1, EGFR |
|  | BP(GO:0048661) | Positive regulation of smooth muscle cell proliferation | 5.089e-9 | JUN, IL6, AKT1, TNF |
|  | BP(GO:0033138) | Positive regulation of peptidyl-serine phosphorylation | 4.823e-10 | HSP90AA1, TNF, AKT1, EGFR, IL6 |
|  | BP(GO:0033135) | Regulation of peptidyl-serine phosphorylation | 6.296e-10 | HSP90AA1, TNF, AKT1, EGFR |
|  | BP(GO:1902808) | Positive regulation of cell cycle g1/s phase transition | 5.841e-7 | CCND1, AKT1, EGFR |
|  | BP(GO:0045428) | Regulation of nitric oxide biosynthetic process | 8.806e-7 | HSP90AA1, TNF, AKT1 |
|  | BP(GO:0034614) | Cellular response to reactive oxygen species | 1.850e-8 | JUN, IL6, AKT1, EGFR |
|  | BP(GO:1900087) | Positive regulation of g1/s transition of mitotic cell cycle | 2.326e-7 | CCND1, AKT1, EGFR |
| Cellular compartments | CC(GO:0097708) | Intracellular vesicle | 0.005488 | EGFR |
|  | CC(GO:0043202) | Lysosomal lumen | 0.04219 | HSP90AA1 |
|  | CC(GO:0005634) | Nucleus | 0.001775 | AKT1, ESR1, JUN, CASP3, HSP90AA1,CCND1 |
|  | CC(GO:0070013) | Intracellular organelle lumen | 0.007287 | IL6, HSP90AA1, EGFR |
|  | CC(GO:0055037) | Recycling endosome | 0.07019 | TNF |
|  | CC(GO:0043231) | Intracellular membrane-bounded organelle | 0.004410 | AKT1, ESR1, JUN, CASP3, HSP90AA1,CCND1 |
|  | \| CC(GO:0005887) \|  \| \| --- \| --- \| | Integral component of plasma membrane | 0.03125 | IL6, TNF, EGFR |
|  | \| CC(GO:0005769) \|  \| \| --- \| --- \| | Early endosome | 0.1253 | EGFR |
|  | CC(GO:0005788) | Endoplasmic reticulum lumen | 0.1337 | IL6 |
|  | CC(GO:0015630) | Microtubule cytoskeleton | 0.1537 | AKT1 |
| Molecular functions | MF(GO:0030911) | TPR domain binding | 0.002498 | HSP90AA1 |
|  | MF (GO:0001091) | RNA polymerase II general transcription initiation factor binding | 0.002498 | ESR1 |
|  | MF (GO:0005138) | Interleukin-6 receptor binding | 0.003495 | IL6 |
|  | MF (GO:0097199) | Cysteine-type endopeptidase activity involved in apoptotic signaling pathway | 0.004990 | CASP3 |
|  | MF (GO:0004709) | MAP kinase activity | 0.005488 | EGFR |
|  | MF (GO:0005164) | Tumor necrosis factor receptor binding | 0.005985 | TNF |
|  | MF (GO:0097200) | Cysteine-type endopeptidase activity involved in execution phase of apoptosis | 0.006482 | CASP3 |
|  | MF (GO:0043325) | Phosphatidylinositol-3,4-bisphosphate binding | 0.01293 | AKT1 |
|  | MF (GO:0032813) | Tumor necrosis factor receptor superfamily binding | 0.01392 | TNF |
|  | MF (GO:0016538) | Cyclin-dependent protein serine/threonine kinase regulator activity | 0.02179 | CCND1 |

**Table S1.** Gene ontology-based bioactivities regulated by hub genes.

| **S.No.** | **Signaling pathways** | **p-value** | **Hit in Query list** |
| --- | --- | --- | --- |
|  | IL-17 signaling pathway | 5.093e-10 | JUN, TNF, CASP3, IL-6, HSP90AA1 |
|  | Pathways in cancer | 1.006e-11 | JUN, TNF, CASP3, IL-6, HSP90AA1, CCND1, AKT1, ESR1, EGFR |
|  | Estrogen signaling pathway | 3.435e-9 | JUN, AKT1, ESR1,EGFR,HSP90AA1 |
|  | FoxOsignaling pathway | 3.580e-7 | AKT1,EGFR,CCND1, IL6 |
|  | JAK-STAT signaling pathway | 8.386e-7 | AKT1,EGFR,CCND1, IL6 |
|  | PI3K-Akt signaling pathway | 3.957e-7 | AKT1,EGFR,CCND1, HSP90AA1,IL6 |
|  | Toll-like receptor signaling pathway | 1.414e-7 | JUN, AKT1, IL6,TNF |
|  | mTOR signaling pathway | 0.002545 | AKT1, TNF |
|  | VEGF signaling pathway | 0.02912 | AKT1 |
|  | Ras signaling pathway | Ras signaling pathway | AKT1, EGFR |

**Table S2.** Role of hub genes in signaling pathways as analyzed by KEGG.
